# Clinical supervision strategies, learning, and critical thinking of nursing students

**DOI:** 10.1590/0034-7167-2022-0691

**Published:** 2023-10-09

**Authors:** Angélica Oliveira Veríssimo da Silva, António Luís Rodrigues Faria de Carvalho, Rui Marques Vieira, Cristina Maria Correia Barroso Pinto

**Affiliations:** I Universidade de Aveiro, Departamento de Educação e Psicologia. Aveiro, Portugal; II Escola Superior de Enfermagem do Porto. Porto, Portugal

**Keywords:** Thinking, Clinical Decision Making, Nursing Students, Learning, Nursing Education., Pensamiento, Toma de Decisiones Clínicas, Estudiantes de Enfermería, Aprendizaje, Educación en Enfermería., Pensamento, Tomada de Decisão Clínica, Estudantes de Enfermagem, Aprendizagem, Educação em Enfermagem.

## Abstract

**Objective:**

To identify the supervisory strategies that Nursing students consider facilitators of the development of critical thinking skills in clinical teaching.

**Methods:**

This is a qualitative study, within the interpretative paradigm, using the focus group methodology. Eight undergraduate nursing students participated in the study.

**Results:**

Participants recognized the indispensability of critical thinking for professional responsibility and quality of care and highlighted the importance of using supervisory strategies adapted to their needs, learning objectives, and the context of clinical practice.

**Final considerations:**

This study highlights the urgent need to establish, within the Nursing curricula, clinical supervision strategies that promote critical thinking and favor the development of skills for good clinical judgment, problem solving, and safe, effective, and ethical decision-making.

## RESUMO

**Objetivo:**

Identificar as estratégias supervisivas que os estudantes de Enfermagem consideram facilitadoras do desenvolvimento das capacidades de pensamento crítico no contexto de ensino clínico.

**Métodos:**

Estudo de natureza qualitativa, inserido no paradigma interpretativo, com recurso à metodologia *focus group*. Participaram do estudo oito estudantes do curso de licenciatura em Enfermagem.

**Resultados:**

Os participantes reconheceram a imprescindibilidade do pensamento crítico para a responsabilidade profissional e qualidade na assistência; e destacaram a importância da utilização de estratégias supervisivas adequadas às suas necessidades, aos objetivos de aprendizagem e ao contexto da prática clínica.

**Considerações finais:**

Este estudo sobreleva a premência em se estabelecer, dentro dos currículos do curso de Enfermagem, estratégias de supervisão clínica promotoras do pensamento crítico, que favoreçam o desenvolvimento de capacidades para o bom julgamento clínico, resolução de problemas e tomada de decisão segura, eficaz e ética.

## RESUMEN

**Objetivo:**

Identificar las estrategias de supervisión que los estudiantes de Enfermería consideran facilitadoras del desarrollo de las capacidades de pensamiento crítico en el contexto de enseñanza clínica.

**Métodos:**

Estudio de naturaleza cualitativa, inserido en el paradigma interpretativo, con recurso a la metodología *focus group*. Participaron del estudio ocho estudiantes del curso de licenciatura en Enfermería.

**Resultados:**

Los participantes reconocieron la imprescindibilidad del pensamiento crítico para la responsabilidad profesional y calidad en la asistencia; y destacaron la importancia de la utilización de estrategias de supervisión adecuadas a sus necesidades, a los objetivos de aprendizaje y al contexto de la práctica clínica.

**Consideraciones finales:**

Este estudio sobrepasa la urgencia en establecerse, dentro de los currículos del curso de Enfermería, estrategias de supervisión clínica promotoras del pensamiento crítico, que favorezcan el desarrollo de capacidades para el bueno juicio clínico, resolución de problemas y toma de decisión segura, eficaz y ética.

## INTRODUCTION

The complex challenges that globalization imposes incorporate the need to establish critical thinking (CT) as a pillar today. It is the duty of educational institutions to prepare their students adequately so that they can provide timely and appropriate answers to the diverse and emerging problems that society and patients impose on them^(1)^. Critical thinking, as a set of skills and dispositions, has sparked the interest of the scientific community and of national and international organizations^(2-3)^. In recent years, efforts have been made to incorporate it into the curricula in different educational institutions.

Widely studied in the last decades, CT can be defined as intentional, rational, and reflective thinking, focused on what one should believe and do^(4)^, which results in interpretation, analysis, evaluation, inference, explanation, and self-regulation^(5)^. It is considered essential for the development of the ability to process information and evaluate its plausibility, to clearly argue and defend one’s position, to foresee consequences, to examine the pros and cons in the decision-making process^(6-7)^. Being able to think critically is evidenced as an important predictor of good academic performance^(8)^. Students with high CT skills are capable of organizing their learning, monitor and evaluate their performance^(9)^, and such skills favor successful decision making^(8)^.

Like globalization, health care has also become complex and demanding; nursing has evolved as a profession, a fact that has generated the need for professionals to demonstrate critical-reflexive characteristics in their daily practice^(10)^. Nursing as a profession is configured as the largest workforce in health care, both by the number of professionals and by its proximity to patients and their families, because it is the nursing professionals who ensure 24 hours of patient care. This proximity and all the complexity involved in nursing care, along with technological advances, require skills that allow nurses to provide safe and quality care^(11-15)^.

In the context of health care, CT can be defined as the rigorous, intentional, and result-focused reasoning based on the patient’s needs^(16)^. For the promotion of safe and quality care, nursing students should be trained in a reflective manner to be able to act and respond assertively to the complex tangle of issues that emerge from the clinical context in the face of dynamic, uncertain, unpredictable, and inconstant situations^(15,17)^. Thus, CT skills are considered essential components for professional responsibility and quality in nursing care^(11-12,14,16,18-19)^.

Thinking critically is not innate, so it requires effort and integration of contexts and teaching and learning proposals that favor its evolution^(20)^. For the development of CT skills, it is important to ensure learning environments that enable the active involvement of students^(21)^, since active learning methodologies can stimulate the development of higher order cognitive processes^(12,15,22-23)^. Active learning methodologies are considered the key to success that guarantees students the ability to analyze evidence and proposals, make fair judgments, propose solutions^(22,24-25)^, evaluate their decisions and, if necessary, go back to the start and reconstruct the whole process^(26)^.

Thus, the clinical teaching context constitutes the space of choice for the development of CT skills due to its dynamic, interactive, unpredictable, mediating, facilitating, and enhancing nature of practical learning^(27-28)^. Clinical teaching, performed in health institutions, is characterized by learning in real context, where the student applies theoretical knowledge in practice, that is, it integrates knowing with doing, resulting in a reflective and critical process and in the improvement of clinical skills and competencies that, when properly conducted, culminates in a conscious, critical, and creative student action^(11,29-30)^. Clinical teaching, through the supervisory process, aims to guarantee the student the acquisition of knowledge and the development of skills and attitudes for the performance of autonomous, conscious, and grounded interventions^(29-31)^.

It is possible to state that CT, as a set of skills, can be taught, and the more these skills are trained, the greater the probability of favorable results, which contributes to the training of students capable of providing assertive answers to the complex problems presented^(5,22,32)^. Critical teaching, due to its real-world nature, favors the development of these abilities (professional competencies), since it enables the articulation between theory and practice, enhanced by the adoption of a proactive learning attitude^(29)^. Clinical supervision strategies (CSS) can influence the development of nursing students’ CT skills, which are essential for their performance in care practice.

Currently, there are several studies that address CT issues^(1-6,8,19,32)^ in a general manner and others more directed to nursing^(18,21-22,26)^; however, there are still no studies relating supervision strategies to the development of CT in nursing students. Based on the premise that CT is a fundamental aspect during the critical teaching of these students, the adoption of constructivist teaching strategies that allow them to improve CT learning should be encouraged. In this sense, it is essential to identify the students’ CT skills and the CSSs that most promote their development.

## OBJECTIVE

To identify the supervisory strategies that Nursing students consider facilitators of the development of CT skills in clinical teaching.

## METHODS

### Ethical aspects

This study was submitted to the opinion of the Ethics Committee of a Nursing School in Northern Portugal, and its ethical and methodological aspects were approved. All study participants signed the Informed Consent Form, which guarantees the right to data privacy, anonymity, and confidentiality.

### Theoretical and methodological framework

The theoretical and methodological framework is based on the qualitative approach. This seeks to interpret and understand a reality from the perspective of the actors involved in the process^(33)^.

The qualitative nature, as a theoretical and methodological foundation, enables the unveiling of the undergraduate nursing students’ thought referential about their experience in the critical teaching context, namely to identify the clinical supervision strategies considered by them as facilitators of the development of their CT skills.

### Study Type

This is a qualitative study, inserted in the interpretative paradigm, since it seeks to interpret and understand the meanings of human action from the perspective of those who live it^(33)^, namely undergraduate nursing students in the context of critical teaching. It is an exploratory and cross-sectional study, using the focus group methodology. For the preparation of the manuscript, to ensure rigor and transparency in the writing of the summary of results, this work was conducted and structured based on the Standards for Reporting Qualitative Research (SRQR), whose objective is to establish standards for reporting qualitative research.

The qualitative nature of focus group data collection provides a rich environment for discussion among several participants, as it allows the researcher to gain an in-depth view of different opinions, knowledge, perceptions, feelings, and experiences on a given topic, which may be analyzed later^(34)^. Its objective is to understand the human being and his/her relations with the theme under investigation, by observing the participants’ discourses, behaviors, and reactions^(33)^. Among the benefits of this methodology, we can highlight its quick execution, the ease of being performed nowadays by videoconference, its dynamic character due to flexibility in its conduction, and contact between the participants and moderator^(34)^.

### Study setting

Study carried out in a Nursing School in Northern Portugal, during the months of May to July 2021. The research was carried out with students enrolled in the 2020/2021 academic year of the Bachelor of Science in Nursing (BSN) course.

### Data source

Bachelor of science in nursing students were part of the sample for this study. The eligibility criteria were: active enrollment in the year 2020/2021 and having at least one critical teaching experience, due to familiarity with the critical teaching context and the CSSs. Eight fourth-year BSN students participated in the focus group session.

During the planning phase of data collection, there was concern about how to recruit the students. Thus, the main researcher was allowed to contact the coordinators of the Clinical Practice curricular units (CU), and they, in turn, contacted the students and explained the nature and objectives of the research. To those who expressed interest in participating, the coordinator requested authorization to give their electronic address, and, by this means, a formal invitation was sent to each student. After accepting the invitation, the students received, also by e-mail, the Free and Informed Consent Form to sign and return to the main researcher. A date and time was then scheduled for the focus group session, and the respective link to access the session was sent to all participants.

### Data collection and organization

Due to the pandemic context caused by covid-19 and the restrictions it imposed on educational establishments and health services, data collection was conducted via videoconference, through the Zoom platform, adopting a focus group session format.

To conduct the focus group, a script consisting of a set of open questions was constructed. In its elaboration, fidelity and validity - essential characteristics that determine the quality of any measurement instrument - were considered. Fidelity is a condition that shows if the measurement instrument provides constant values from one use to the next; in this sense, a pre-test of the script was carried out with a focus group composed of four students who met the eligibility criteria for the research and who were not part of the group of participants. After analysis, there was no need for reformulation. Validity was checked through the evaluation of the script by an expert external to the study: a professor and researcher in nursing with 20 years of experience in clinical supervision, who gave a favorable opinion about the instrument.

The execution or moderation of the focus group was carried out by the main researcher, assisted by another researcher of the team, experienced in using the methodology. The session lasted about two hours. The session was recorded in image and audio and was destroyed after being transcribed.

### Analysis of results

Content analysis was used globally, according to the methodology proposed by Bardin. Such analysis allows us to verify the order, intensity, and frequency of a discourse^(33)^, with the aim of improving the results, expanding its validity and, consequently, enabling a reliable final interpretation^(35)^.

Data analysis was performed by two independent researchers, with a backup researcher who was involved in case of disagreement, and followed three phases: organization, coding, and categorization. The first phase comprised the full transcription of the focus group participants’ speeches according to their sequence. To guarantee focus group anonymity, each participant was classified with the letter P (Participant) plus a number corresponding to the order of his or her speech (P1, P2, ...P8). Next, a skimming of the entire document that composed the corpus of analysis was performed, followed by an exhaustive reading. In the second phase, we performed the codification based on the theme and listed according to the frequency and intensity in the speeches. Finally, the semantic and lexical categorization was performed considering the meanings and reasoning of the speeches, based on which the categories and respective subcategories emerged^(35)^.

## RESULTS

Eight BSN students participated in the focus group session, six female and two male. All of them were attending the 4th year of the Clinical Practices CUs course; and their ages ranged from 21 to 24 years, with an average age of 22.

The content analysis of the participants’ discourse allowed us to organize the information into categories and subcategories as shown in Chart 1. The categories found were “Critical thinking skills”, “Factors that facilitate the development of critical thinking”, “Factors that hinder the development of critical thinking”, and “Clinical supervision strategies”.

**Chart 1:** Categories and subcategories of the content analysis of the focus group participants’ discourse

CATEGORY	SUBCATEGORY
Critical thinking skills	Interpretation
	Analysis
	Evaluation
	Inference
	Explanation
	Self-regulation
Factors that facilitate the development of critical thinking	Having a supervisor
	Having multiple supervisors
	Supervisory relationship
	Experience in clinical supervision
	Student’s characteristics
	The role of the supervisor
	Integration into the team
Factors that hinder the development of critical thinking	Nurse’s attitude
	Lack of feedback
	Communication problems
	Not wanting to be supervisor
	Not having a supervisor
	Feeling humiliated
	Lack of support
	Negative experiences
Clinical supervision strategies	Pedagogical questions
	Guidance
	Reflection
	Individualized study
	Support
	Encouraging
	Feedback
	Discussion of clinical cases
	Setting Individual Goals
	Observation
	Encouraging communication with the team

### Critical thinking skills

The participants recognized the importance of CT in the initial training of nurses, listing it as a factor that favors professional responsibility and quality in nursing care, since it promotes reflective thinking, meaningful and autonomous involvement in the complex context of health care, clinical judgment, and the development of decision-making skills.

*I think it’s quite important that we develop our critical thinking because I think that’s one of the foundations of nursing practice; and I think that we as students, the earlier we develop it, the easier it will be for us when we are nurses.* (P1)

[...] *I think it’s a fundamental part of being a good nurse* [...]. (P5)

However, despite the extensive dissemination of the need for CT in nursing education, it is perceived that students are often not stimulated or are even prevented from putting their competencies into practice. Such difficulty is evidenced in their speech:

[...] *it is true, sometimes we enact our critical thinking and we even manage to sustain it, but it is not always used, because many times we are even told “This is the protocol here”, and we actually end up performing the intervention according to the protocol, and not according to what we think, which is even more based on the most current evidence* [...]. (P7)

In addition to recognizing its importance, participants also highlighted that the CT skills (evaluation, interpretation, analysis, inference, explanation, and self-regulation) are essential for their good performance in critical teaching, namely for clinical judgment, decision-making, and problem solving.

*It is basically having the tendency not to say, oh, this is like this, it’s like that, because that person is telling me it’s like that; it’s having the ability to make the decision ourselves, taking into account the data that we have and the information that we can collect from that problem.* (P7)

*Even if there are protocols at the service level, whatever they are, we shouldn’t look at them as a norm or a rule. We should respect them, but take into consideration what principles or what kind of bibliography they are based on... we should always look for evidence that justifies and supports the practices* [...]. (P8)

### Factors that facilitate the development of critical thinking

In the critical teaching context, the relationship between supervisors, students, and multiprofessional team is key to facilitate student learning. The spirit of cooperation favors autonomy, good clinical judgment, and the decision-making process. Regarding facilitating factors for the development of CT, participants highlight the importance of having a supervisor, having multiple supervisors, the supervisory relationship, experience in clinical supervision, student characteristics, the role of the supervisor, and integration into the team.

*The establishment of a good supervisory relationship I think is the key, because from a good supervisory relationship comes all the rest, comes questioning, autonomy, trust, and also availability, which then will encourage us to seek more information, to question more, and learn more about the service we are in.* (P4)

[...] *much more important than the strategies used, is also knowing the role that the supervisor has* [...] *knowing for a fact where he can really contribute to our critical thinking.* (P6)

### Factors that hinder the development of critical thinking

As supervisors move away from the characteristics previously described, they establish an authoritarian, oppressive, hostile, apathetic environment with little communication and make it impossible to create a good supervisory relationship, besides causing negative experiences in students. The absence of a good relationship between supervisor and supervised shows itself as a hindrance to the development of CT.

The analysis of the participants’ discourse allowed us to identify the following aspects as hindering the development of CT: nurse’s attitude, lack of feedback, communication problems, not wanting to be a supervisor, not having a supervisor, lack of support, and negative experience.

[...] *I did many shifts with this supervisor, and she had a very authoritarian and very hostile attitude* [...] *I think I could have learned a lot more, and I didn’t learn exactly because of this attitude of hostility and neglect.* (P3)

[...] *there was a little bit of a mocking attitude towards me for not knowing, and “how is it possible that you don’t know this?*” (P3)

[...] *my family was worked up, everything was going wrong, and the supervisor* [...] *never showed any concern, not even on a knowledge level, not on a personal level, nothing. It was the same thing being with her, I did things with her, and if I wasn’t with her it was the same, that was it.* (P4)

*Having been placed with a supervisor who didn’t want to have students, I know it’s a little contradictory, but she told me, in this case directly, that she didn’t want to have students; and I think that makes clinical teaching a little difficult, because the person in particular didn’t ask me questions, when I asked questions she answered me in a less than friendly way, or sometimes she didn’t answer me at all. And then, throughout the clinical teaching, it felt a little bit as if the person was doing me a favor, and I didn’t feel secure and confident to be able to develop what I wanted, and I think it hindered my learning a little bit.* (P1)

### Clinical supervision strategies

The strategy adopted by the supervisor is established as the structuring axis of the teaching and learning process within critical teaching, since it allows students to develop essential clinical skills aiming at success in their performance. Regarding the CSSs, the participants highlight the pedagogical questions, reflection, individualized study, guidance, support, encouragement, feedback, discussion of clinical cases, definition of individual goals, observation and stimulation, and communication with the team.

[...] *the fact that the supervisors ask us questions helps us to reflect on certain issues that are important and that, perhaps, we would not pay much attention to.* (P2)

[...] *the fact that those questions, which are questions of another level, that ultimately take us further, are those questions that are not basic, but that we have to study in depth and understand different relationships to answer those questions* [...]. (P8)

[...] *the questions, I think are important, but I think the moment, that is, when they ask us the questions is fundamental; I just don’t think it should be a stressful moment* [...]. (P5)

*It really made me reflect and think* [...]. (P1)

[...] *that is, with one question, I think I could encompass great knowledge, which would then have repercussions on my critical thinking in the short or long term.* (P7)

[...] *but also, at the same time, it would guide my study at the initial moment; it would help, for example, “Look, right now, I think it makes sense for you to study this.”* (P3)

[...] *she was a supervisor who cared about me as a person. For example, if there was any change, she was the first to say: “If you can’t make it, you can come and do it on another day; there’s no problem.* (P3)

[...] *in more stressful situations, she was the first one to say: “Calm down, I’ll help you, I’m here and you can do it”.* (P3)

*I consider feedback as something central, that is, to understand how well we are doing in clinical teaching, because we often have a wrong perception* [...]. *I think we should always understand how we are doing and what we can improve on.* (P8)

[...] *and I think that, in the third year, in some of the initial clinical teaching, it was essential to establish objectives* [...], *also because when we start clinical teaching, we somehow feel lost, because there are a lot of things that we must know, so the definition of objectives ends up guiding us in some way.* (P8)

The participants recognized the indispensability of CT for clinical practice, describing it as essential for professional responsibility and quality in nursing care, by virtue of being an enabling factor for reflective thinking, autonomy, good clinical judgment, acquisition of problem-solving skills, and safe, effective, and ethical decision-making. In addition, they highlighted the importance of establishing a good supervisory relationship, i.e., the need for the supervisor to get to know the student within his or her personal and academic journey. This close relationship was considered essential in determining the supervisory strategy best suited to the students’ needs, learning objectives, and clinical teaching context.

## DISCUSSION

Peter Facione, through the memorable The Delphi Report, emphasizes the indispensability of the development of CT capabilities for the execution of sensible judgments and correct decisions. The abilities, also recognized as cognitive competencies or skills, are classified into six spheres: interpretation, analysis, evaluation, inference, explanation, and self-regulation^(5-6,36)^. These competencies are considered primordial for the student’s good academic, personal, and professional performance^(36)^ since they allow them to improve problem-solving skills and, in turn, make assertive decisions^(9)^.

The participants’ discourse makes it possible to identify the critical thinker as one updated in knowledge, diligent in the search for a valid reason, thoughtful, flexible in his judgments, able to judge the credibility of information. However, it can be inferred that the absence of a cordial relationship between supervisor and supervised as well as the impossibility of putting current scientific knowledge into practice constitute major hindrances to the development of the students’ CT. Similarly, the critical thinker is described as curious, well-informed, creative, open to different possibilities, possessing reflective thinking, and therefore capable of judging the credibility of information^(17)^. Because the context of health care demands prudence from the nursing student in the search for and selection of information^(10)^, it becomes urgent to develop the habit of questioning and searching for scientific evidence to support safe and quality care.

Critical teaching allows the student to experience valuable moments of observation and interaction, as it favors the development of essential knowledge, skills, and attitudes for professional practice, provides self-regulation resources, besides facilitating the appropriation, mobilization, and materialization of knowledge^(29)^. In this environment, the supervisory process is configured as dynamic, interactive, facilitator, and which empowers learning, since it is based on a relationship of trust and mutual help, in which the supervisor adopts strategies for the personal and professional development of those supervised^(37)^.

The clinical supervisor, as the mediator of the students’ learning and professional development, ensures the transition from being a student to being a professional^(29)^. In this framework, the supervisory strategy constitutes an important phase in the training process since it provides the planning of actions and the organization of activities that favors students’ personal and professional development^(37)^.

Anchored in the principle of equity, supervisors should not treat students in an exactly equal manner regarding learning, a fact that is justified by the individual needs inherent to each supervisee^(38)^. There is no single, incontestable strategy that fits all contexts and all students; rather, there are different strategies that can be used in different combinations. For the strategy selection to be successful, it is important to consider the clinical context, the students, the learning objectives, and the available resources, which is why individualization of teaching is indispensable^(37)^.

Reflection allows students to construct and appropriate knowledge through their professional practice. In this process, the supervisor has a fundamental role in leading the student in the reflective process, either by creating real learning opportunities, or by provoking him/her in the search for more knowledge. Upon a doubt or difficulty, reflective thinking is initiated, followed by a mental process of questioning, which motivates the student to research so that he can then resolve that doubt or difficulty^(39)^. Reflection improves the capacity to solve problems and makes it possible to revisit and reformulate the experience lived, with the objective of establishing alternatives and constructing new learning^(37)^. The reflective process is enunciated as an effective strategy for the promotion of CT^(17)^, since reflecting on priorities in care promotes the development of confidence, autonomy, and communication skills^(28)^.

To formulate questions that instigate students’ restlessness and motivate them in the search for answers is pointed out as a propulsive strategy for reflective and creative thinking^(37)^. As the questions increase the students’ interest and curiosity, they contribute to experiential learning, enabling the connection between knowledge and practice^(17)^. At the same time, the supervisor, at opportune moments, in a friendly manner and withholding his/her evaluative character, asks questions. Besides facilitating the construction of a competent professional, it also instills in the student the habit of asking questions, allowing progress toward the potential development of CT skills.

Providing feedback allows students to become active subjects in the teaching and learning process, since it facilitates the self-evaluation process, and reflective, creative, and critical thinking^(40)^, and autonomy^(17)^, characteristics that are indispensable to nurses. Thus, the supervisor should find the most opportune moment to provide effective, clear, and constructive feedback^(40)^.

### Study limitations

There are two limitations. First, the participants are only 4th year BSN students, i.e., graduates; this made it impossible to determine the strategies that promote CT for all BSN students in a critical teaching context. The second limitation relates to the fact that the study was conducted in a pandemic context, so online data collection did not allow establishing a physical meeting place with the participants, a fact that may have conditioned the responses due to the lack of familiarity with this format. In this sense, it is suggested to carry out studies in post-pandemic contexts, with students at different stages of critical teaching, in the early and final academic years, which will make it possible to demonstrate the causal relationship between the CSSs and the CT skills in students throughout the Nursing course.

### Contributions to the field

The study highlights the need for the use of CSSs that favor the reflective process. It also reiterates the urgency to establish within the curricula of undergraduate nursing courses constructivist teaching strategies that allow students to develop CT, essential skills for good clinical judgment, problem solving, and safe, effective, and ethical clinical decision-making.

## FINAL CONSIDERATIONS

Students recognized the importance of CT skills in the critical teaching context, considering them fundamental for clinical judgment, decision making, and problem solving. Since critical thinking demands effort and association of contexts and strategies that enable its development, this study highlights the importance of using different CSSs. Thus, the perspective of strategies acting in interchangeable and complementary ways is emphasized. Pedagogical questions, reflection, individualized study, orientation, support, encouragement, feedback, discussion of clinical cases, definition of individual goals, observation, and stimulation of communication with the team are the strategies identified as the most favorable for CT. At certain times, it is appropriate to use several strategies at the same time; at other times, only one is sufficient to achieve the learning objectives. To establish the most appropriate supervisory strategy for the student and the learning context, it is essential that the supervisor, in addition to knowing the academic path of his supervisee, recognize his individuality and needs.

## Data Availability

https://doi.org/10.48331/scielodata.PC3XOX
